# State and situation of avian influenza in the Eastern Mediterranean Region

**DOI:** 10.1111/irv.13137

**Published:** 2023-04-23

**Authors:** Rebecca Badra, Abdinasir Abubakar, Stefano Tempia, Noore Alam, Hala Abou ElNaja, Ghazi Kayali, Wasiq Khan

**Affiliations:** ^1^ Human Link DMCC Dubai UAE; ^2^ Infectious Hazard Prevention and Preparedness, WHO health Emergencies Programme, WHO Regional Office for the Eastern Mediterranean Cairo Egypt

**Keywords:** avian influenza, Eastern Mediterranean Region, One Health, outbreaks, research, surveillance

## Abstract

Avian influenza viruses have had a significant burden of disease on animal and public health in countries of the Eastern Mediterranean Region. In this review, we aimed at describing the state of avian influenza in the region from 2011 to 2021. We gathered information available through the peer‐reviewed scientific literature, public gene sequence depositories, OIE World Animal Health Information System platform, World Health Organization FluNet, Joint External Evaluation reports, and governmental, Food and Agriculture Organization of the United Nations, and World Organization for Animal Health websites. We used an interdisciplinary perspective consistent with the One Health approach to perform a qualitative synthesis and making recommendations. Analysis showed that although avian influenza research in the Eastern Mediterranean Region has gained more attention during the last decade, it was limited to only few countries and to basic science research. Data highlighted the weakness in surveillance systems and reporting platforms causing underestimation of the actual burden of disease among humans and animals. Inter‐sectoral communication and collaboration for avian influenza prevention, detection, and response remain weak. Influenza surveillance at the human‐animal interface and the application of the One Health paradigm are lacking. Countries' animal health and public health sectors rarely publish their surveillance data and findings. This review suggested that surveillance at the human‐animal interface, research, and reporting capacities should be enhanced to improve understanding and control of avian influenza in the region. Implementing a rapid and comprehensive One Health approach for zoonotic influenza in the Eastern Mediterranean Region is recommended.

## 1 INTRODUCTION

Avian influenza (AI) viruses cause devastating outbreaks in poultry with severe economic consequences due to high mortality in birds and significant drop in egg production.^1^ Based on pathogenicity, AI viruses are classified into highly pathogenic avian influenza (HPAI) viruses that cause severe disease in poultry and result in high death rates and low pathogenic avian influenza (LPAI) viruses that cause mild disease in poultry.^2^ Eastern Mediterranean Region (EMR) countries lie under four of the eight global migratory bird flyways^3^: Central Asia–India, West Asia–Africa, Mediterranean–Black Sea, and East Atlantic. This opens the door to the transmission of AI viruses from migratory birds to the resident wild birds, domestic poultry, mammalian species, and humans.

Since 2003, H5N1 spread has been responsible for millions of poultry infections and several human outbreaks and deaths in many countries in Asia, Europe, the Middle East, and Africa,^4^ and has become endemic in poultry populations in some countries (Bangladesh, China, Egypt, India, Indonesia, and Vietnam).^5^ H5N1 AI has spread rapidly through the EMR in 2006 with large epizootics reported in Iraq, Egypt, Jordan, Palestine, Afghanistan, Pakistan, Djibouti, and Sudan.^6^ The burden became more significant in the EMR when transmission of H5N1 from infected birds to humans was confirmed in Iraq, Egypt, Djibouti, and Pakistan starting 2006.^6^ From 2003 to 15 April 2021, 862 confirmed human cases for avian influenza H5N1 and 455 fatalities have been reported to WHO globally, out of which 366 confirmed human cases and 123 fatalities reported from EMR,^7^ all of whom were exposed to sick or dead poultry. Egypt, where the disease remains endemic, has reported the most cases in the EMR, with a total of 359 reported human cases and 120 deaths (33.4% CFR).^7^ Since its emergence and spread in some EMR countries, the H5N1 virus has evolved resulting in a range of circulating clades. HPAI H5N1 clade 2.2.1 circulated for over a decade in Asia, Africa, and the Middle East. The emergence of clade 2.3.4.4 H5Nx resulted in a significant global spread of the virus.^8^ In Africa, Egypt was the first country to detect HPAI in 2005 in a common teal. Since its introduction, the virus became endemic in poultry and diversified into clade 2.2.1 and sub‐clades 2.2.1.1a and 2.2.1.2.^8^ Clade 2.2.1.2 viruses established themselves in Egyptian poultry until they were eventually replaced by the wider circulation of H9N2 viruses and the sporadic introduction of clade 2.3.4.4 H5 viruses.^9^ In 2016, a HPAI H5N8 virus of clade 2.3.4.4 was first isolated from the common coot from Egypt and was closely related to H5N8 viruses from Europe suggesting its introduction into Africa through migratory birds.^10^ Since then, several cases of H5N8 have been recorded among domestic poultry in Egypt.^11^ Sporadic outbreak of H5N1 2.3.2.1c clade virus occurred in Lebanon in poultry in 2016, and genome analysis showed clustering with viruses that were reported in Europe and West Africa.^12^

Avian influenza viruses of the H9N2 subtype are widely circulating in avian species causing severe economic losses. H9N2 is endemic in poultry of many EMR countries and has been reported in Lebanon, Jordan, Egypt, Tunisia, Saudi Arabia, and the UAE.^13^ Therefore, avian‐to‐human transmission becomes an important public health concern as H9N2 viruses have the ability to cross the species barrier and infect humans.^14^ The apparent adaptation of avian H9N2 virus to mammalian cells is in agreement with the WHO's alertness for a possible public health threat. Sero‐epidemiologic studies from different EMR countries documented evidence of H9N2 antibodies in exposed humans.^15^ Few cases of infection to humans were reported from Egypt. H9N2 viruses are likely enzootic in all EMR countries^16^, ^17^ and have undergoing genetic evolution due to transmission, including vaccinations,^18^ as H9N2 vaccines are used in some EMR countries such as Egypt, Lebanon, and Jordan. Within country, evolution, and emergence of reassortants and new subclades were reported.^19^

We conducted this systematic review to describe the state and situation of avian influenza in the EMR over the period 2011–2021.

## 2 METHODS

This study was designed following the Preferred Reporting Items for Systematic Reviews and Meta‐Analyses (PRISMA) 2020 for review of the published peer reviewed literature.

We systematically reviewed many sources, including the OIE World Animal Health Information System (OIE‐WAHIS) website and extracted data on reported AI outbreaks in animals. We searched the WHO website and Flunet (Global Influenza Programme (who.int]) for reported AI cases in humans. We searched the influenza Research Database (IRD) (www.fludb.org) to understand the status of influenza sequence reporting from EMR countries. We expanded the search to include the Joint External Evaluation (JEE) reports of EMR countries, governmental, Food and Agriculture Organization of the United Nations (FAO), and World Organization for Animal Health (OIE) websites. We focused on the analysis of surveillance platforms for AI within the human and animal health sectors.

### 2.1 Search strategy

We used PubMed to search the peer‐reviewed literature using the search terms “Avian Influenza” and “country name” (Figure S1). Countries of the EMR region include Afghanistan, Bahrain, Djibouti, Egypt, Iran, Iraq, Jordan, Kuwait, Lebanon, Libya, Morocco, Oman, Pakistan, Palestine, Qatar, Saudi Arabia, Somalia, Sudan, Syria, Tunisia, United Arab Emirates (UAE), and Yemen.

### 2.2 Eligibility criteria

The included publications met the following eligibility criteria: (1) publications report avian influenza research in one or more EMR countries; (2) publications are published in the period from 2011 to 2021; (3) publications are assigned to one of the following categories: virology, editorial, epidemiology, epizootiology, health care management, modeling, reporting, review, surveillance. Publications were excluded for the following reasons: (1) duplicates, (2) records not related to avian influenza research in the EMR.

### 2.3 Data extraction

Publications from PubMed search were exported to Endnote X8 library. Duplicates were removed. Retained publications were exported to an excel spreadsheet for abstract revision. Records not related to avian influenza research in the EMR were excluded. The following data were extracted from each included study: publication year, country, and category.

## 3 RESULTS

### 3.1 Key ecological and epidemiological findings

In the past decade, 10,095 outbreaks of avian influenza were reported in avian species in the region. Between 2011 and 2016, the majority of the reported outbreaks were related to HPAI H5N1 viruses from clade 2.2.^20^ Additionally, the H9N2 virus was first reported in Egypt in 2011 and later detected in other countries in North Africa. In 2016, H5 clade 2.3.4.4 was detected in the region and together with H9N2 replaced the H5N1 of clade 2.2. HPAI H5N8 continues to be detected sporadically in the region. Investigations showed that viruses were introduced through infected wild migratory birds, but in general, wild bird surveillance is limited in the EMR.

The burden of disease in the animal health sector is high whether HPAI or LPAI H9N2 viruses are circulating. HPAI causes high mortality rates in infected domestic poultry while H9N2 causes severe losses in egg production among layer flocks and decreases the immunity of infected birds allowing for severe secondary infections by other pathogens. In the EMR, chickens constitute the majority of raised poultry hence the burden of disease is highest amongst this species. Nonetheless, there is evidence of other domestic bird species such as pigeons, geese, quails, partridges, and turkeys being infected with HPAI and LPAI viruses.

The shift in the ecology of AI in the region led to a shift in the disease burden in humans. HPAI H5N1 was responsible for the majority of human infections. As this was replaced with clade 2.3.4.4 viruses, no human infections with HPAI were reported in the EMR. However, some human infections with H9N2 viruses were reported in Egypt but all were mild.

Comparing human case reports with available serological studies reveals that current surveillance systems are not reflecting the true incidence and burden of disease.^4^ For instance, data from a cohort study of Egyptian poultry growers conducted between 2015 and 2019 revealed that 11% of poultry growers had neutralizing antibodies against H9N2 and negligible seroprevalence of H5N1.^15^ This finding is in line with a previous study suggesting that the public health surveillance systems are only detecting severe cases of HPAI infections that are admitted to hospitals.^21^ Further research indicated that human infections with HPAI and LPAI viruses may be mild to moderate with symptoms resembling seasonal influenza viruses supporting the notion that surveillance systems as they stand tend to miss those infections.^22^ Research indicates that the cause of human infections with AI viruses is exposure to sick or dead domestic poultry.^23^ However, the true routes of transmission were not studied.

### 3.2 Avian influenza virus outbreaks in animals

**FIGURE 1:**
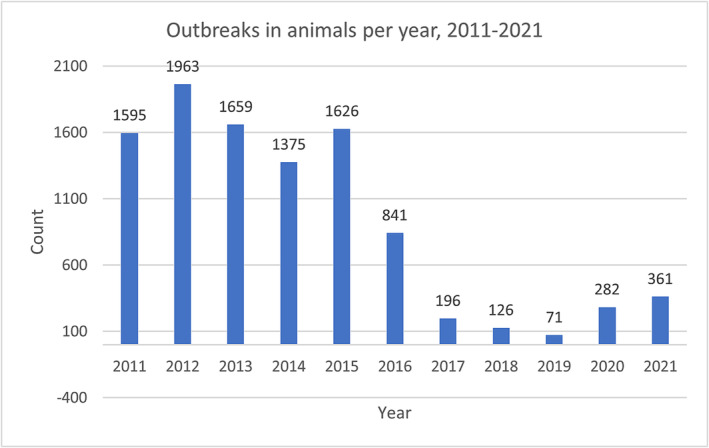
AI virus outbreaks in animals per year.

In several EMR countries, H5N1 and/or H9N2 viruses are endemic in the poultry populations. Because avian influenza viruses are in continuous evolution and considering their potential to cross species barrier and infect humans, continuous systemic surveillance is of utmost importance for both veterinary health and public health. Outbreak reporting from EMR was almost continuous between 2011 and 2015 and decreased from 2016 to 2019. This could be explained by the actual decrease of AI outbreaks, the animal health not reporting all AI outbreaks to OIE‐WAHIS, or the animal health not detecting all AI outbreaks due to lack of technical and/or financial means. Reporting AI virus outbreaks in animals increased slightly in 2020 and 2021 (Figure 1). This might be caused by the stakeholders noticing the importance of reporting outbreaks to prevent potential future pandemics similar to COVID‐19. Iran was the most affected country with 8228 recorded outbreaks in animals from 2011 to 2021 followed by Egypt and Iraq (Table 1). Out of the 22 EMR countries, 11 (50%) did not report any AI virus outbreak in animals from 2011 to 2021, three (13.6%) countries reported less than 10. These modest numbers could be an underestimation of the actual burden of disease. Updating surveillance programs and reporting systems should be considered to reflect the real AI virus transmission among poultry. Almost all the outbreaks were reported in domestic poultry, out of which 15% were HPAI and 85% were LPAI which is most likely due to H9N2 (Figure S2).

**TABLE 1:** AI virus outbreaks in animals by country 2011–2021.

Country	Total	HPAI	LPAI
Afghanistan	7	7	0
Egypt	1310	1310	0
Iraq	395	40	355
Iran	8228	119	8109
Kuwait	11	11	0
Lebanon	2	2	0
Libya	20	2	18
Pakistan	48	8	40
Palestine	38	14	24
Saudi Arabia	35	35	0
Tunisia	1	1	0
Total	10,095	1549	8546

### 3.3 Avian influenza A(H5N1) virus infections in humans

**FIGURE 2:**
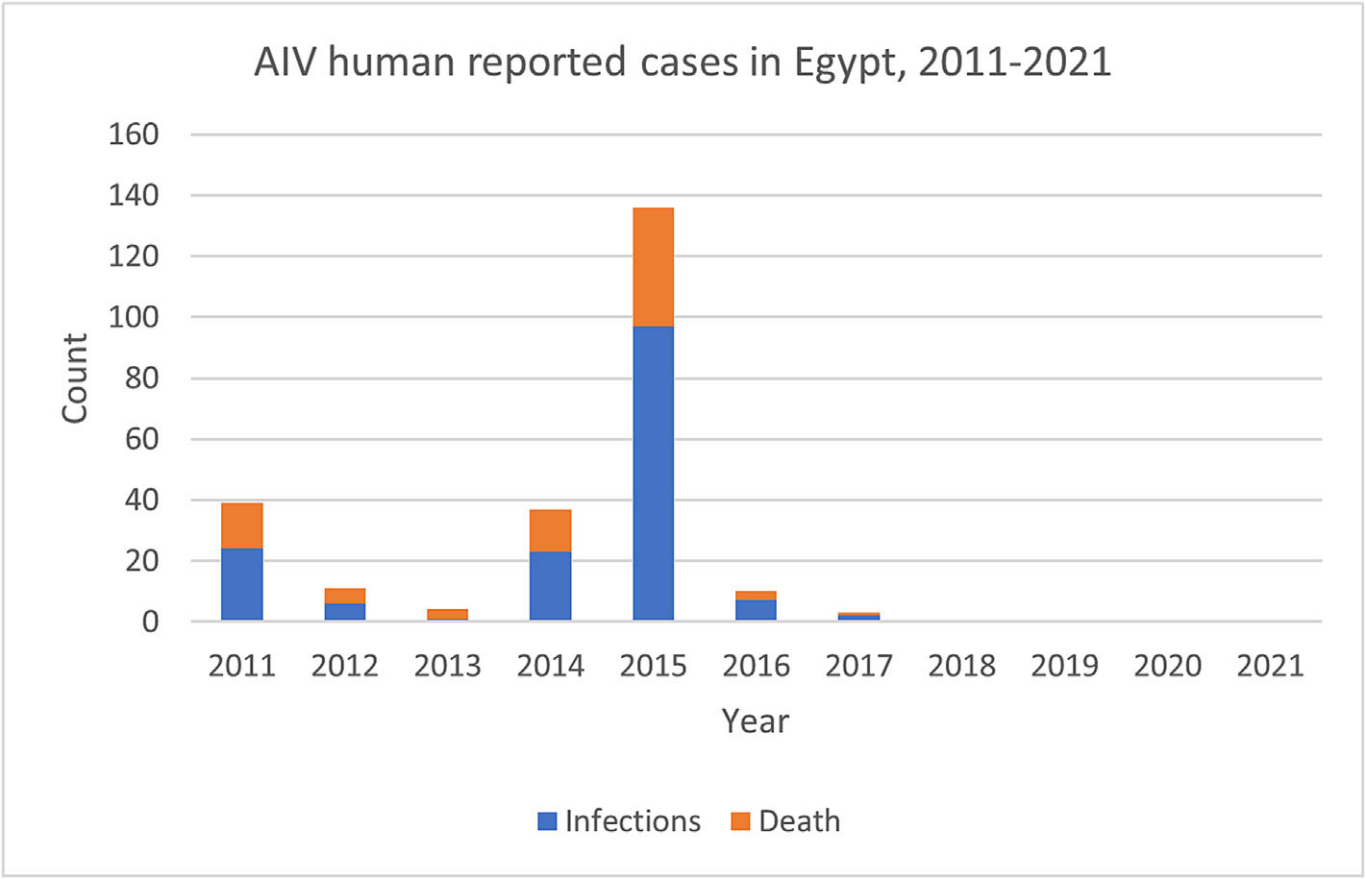
AI virus human reported cases in Egypt by year.

In the EMR, not all countries report AI surveillance data. Between 2011 and 2021, only Egypt reported human cases of AI H5N1 infections. The cumulative number of human cases for A(H5N1) reported to WHO from Egypt was 240 cases including 80 deaths with a peak of 136 cases including 39 deaths in 2015 (Figure 2). The last AI virus cases from EMR countries were reported in 2017. It is likely that human AI infections are occurring more frequently than reported especially if the disease is asymptomatic or mild as is the case of human infections with H9N2 viruses that are enzootic in the region. This highlights the weakness in surveillance systems of EMR countries and the necessity of updating surveillance programs to monitor influenza activity in human populations and the need for coordinated efforts among veterinary and public health sectors under a One Health framework.

### 3.4 Published literature

A total of 540 relevant records were retained. AI research in the EMR has gained more attention during the last decade. Publications increased since 2011 and reached 540 research publications (Figure S3). The majority of AI publications in the EMR are from Egypt, Iran, Iraq, Pakistan, and Saudi Arabia (Figure S4). Publications from Egypt alone account for more than 54% of the published AI research in the region. Iran followed with 104 publications and Pakistan with 56 publications. Research on AI virus from Saudi Arabia and Tunisia were modest with 21 and 16 publications respectively. The research activity in the remaining EMR countries remained minimal. Basic science research focusing on virology (67.7%) were the most frequently published types of work (Figure S5). Within virology, researchers concentrated their work on studying the antigenic, molecular aspects and characteristics of avian influenza viruses in their host animals. Active and passive surveillance (10%) at the human‐animal interface were also of interest for researchers to determine the dynamics of circulating avian viruses and their transmission among species. Few publications focused more on vaccines development and vaccine model testing.

### 3.5 Avian influenza virus sequences

Searching the Influenza Research Database (IRD) for published AI sequences since 2011 yielded 3398 sequences. A surge in submitting sequences was observed in 2019 with 754 sequences (Table 2). The submission rate dropped considerably to 120 and 9 sequences in 2020 and 2021, respectively. This may be explained by the fact that researchers have shifted their interest from AI virus research towards COVID‐19 research. Furthermore, all funds and resources were mobilized for COVID‐19 research.^24^ The largest proportion was from Egypt. Egypt is the most affected by AI virus and researchers are making serious efforts in performing AI virus research (Figure S6). This explains the fact that Egypt leads AI virus research activities in EMR, contributing to more than 54% of publications and more than 63% of submitted AI virus sequences. Pakistan contributed 682 sequences and Saudi Arabia 111 sequences. Other EMR countries published few to no sequences. Equally, partial and complete genome sequences were submitted to GenBank, showing that researchers are generating sequences for research and not just for diagnostics. Sequences were mostly from domestic birds (73%), of H5 and H9 subtypes (Figure S7), endemic in reporting countries, and of HA and NA proteins (Figure S8).

**TABLE 2:** AI virus sequence submission by country and by year.

Country	2011	2012	2013	2014	2015	2016	2017	2018	2019	2020	2021	Total
Egypt	226	102	342	505	138	357	116	53	307	15	1	2162
Iraq	0	0	1	0	0	0	0	0	10	0	0	11
Iran	12	59	57	6	0	0	0	40	0	0	0	174
Jordan	0	0	1	0	0	0	0	88	0	0	0	89
Kuwait	0	0	1	0	0	0	0	0	0	0	0	1
Lebanon	16	0	1	0	0	8	0	3	0	0	0	28
Libya	0	0	1	0	0	0	0	0	0	0	0	1
Morocco	0	0	0	0	0	0	17	24	0	0	8	49
Pakistan	36	79	7	33	29	0	26	29	435	8	0	682
Saudi Arabia	3	2	7	0	0	0	0	0	2	97	0	111
Tunisia	1	0	0	3	9	1	0	0	0	0	0	14
United Arab Emirates	8	3	39	21	0	5	0	0	0	0	0	76
Total	302	245	457	568	176	371	159	237	754	120	9	3398

### 3.6 Zoonotic influenza surveillance platforms in the Eastern Mediterranean Region

In countries with better surveillance capacities, AI surveillance is either included in the Severe Acute Respiratory Illness/Influenza‐Like Illness (SARI/ILI) surveillance systems or is event‐based upon reports of outbreaks from the animal health sectors. Event‐based surveillance that is tied to reports from the animal health sector is weak as our findings suggest that surveillance capacities in the animal health sector are generally less‐developed than their human health counterparts. Inter‐sectoral communication and collaboration for AI prevention, detection, and response remains weak. Influenza surveillance at the human‐animal interface and the application of the One Health paradigm are lacking. There was no indication on complementary coordinated efforts to enhance AI and other zoonotic disease surveillance in the region by the WHO, FAO, and OIE. The main focus of the WHO has been SARI/ILI surveillance with focus on seasonal influenza viruses. The FAO developed some projects to enhance surveillance capacities within the animal health sectors. The OIE maintains the platform to report AI outbreaks in animals and conducts country assessments of the performance of veterinary services, yet those reports are not publicly available. Countries' animal health and public health sectors rarely publish their surveillance data and findings. Similarly, surveillance‐related research publications represent only 10% of published AI research papers from 2011 till 2021.

### 3.7 Joint External Evaluation's analysis of zoonoses surveillance systems of Eastern Mediterranean Region countries

Reviewing published Joint External Evaluation (JEE)’s reports of 17 EMR countries (Afghanistan 2017, Bahrain 2017, Djibouti 2018, Iraq 2019, Jordan 2017, Kuwait 2018, Lebanon 2017, Libya 2019, Morocco 2017, Oman 2017, Pakistan 2016, Qatar 2017, Saudi Arabia 2017, Somalia 2017, Sudan 2017, Tunisia 2017, and the UAE 2017) showed that AI virus is an important disease discussed in their reports and listed by the animal and/or public health sectors of most EMR countries among priority zoonotic diseases. Some of EMR countries have established passive and/or active surveillance systems for priority diseases. Zoonotic diseases are reported by the public health and animal health sectors of some EMR countries to the WHO and OIE as part of the list of notifiable diseases. JEE's reports highlighted weaknesses and challenges related mainly to the lack of technical expertise and diagnostic laboratory capacities for zoonotic diseases detection and response in the veterinary and/or public health sectors, absence of multisectoral sharing of surveillance data, absence of joint surveillance systems between the animal and public health sectors, and absence of formal structures or framework to facilitate and sustain multisectoral collaboration.

### 3.8 FluNet

Reviewing virologic data available on FluNet between 2011 and 2021 (WHO FLUMART OUTPUTS) (Figure S9) revealed that the following EMR countries were contributing data: Afghanistan, Egypt, Iran, Iraq, Jordan, Lebanon, Morocco, Oman, Pakistan, Qatar, Saudi Arabia, Syria, Tunisia, and United Arab Emirates. However, reviewing SARI surveillance reports from 2011 to 2021 in EMR countries show that it was capable of detecting different subtypes of influenza virus except for zoonotic influenza. From 2011 to 2021, Influenza subtype H5 was only reported by SARI surveillance in Egypt (Figure S10).

## 4 DISCUSSION

EMR countries must remain vigilant and aware of the threat of HPAI H5Nx viruses as they continue to evolve and circulate globally. H9N2 viruses are enzootic in most countries of the EMR. Although this virus does not cause a severe disease in poultry, it is considered a pathogen of high economic consequences especially in layer and breeder flocks. AI risk assessment exercises must be routinely conducted on country and regional levels using available WHO tools and platforms with a multi‐sectoral audience.

In the region, live bird markets and backyard poultry growing is common. Those production sectors tend to have low biosecurity measures and are likely the hotspots for AI activity. Enhancing biosecurity within those sectors and increasing awareness of market vendors and backyard growers may assist in mitigating the risk of AI.

Research further indicates that spill‐over of H9N2 from poultry to humans occurs more frequently than what the surveillance systems detect and report. The burden of AI among exposed humans can be better understood through population‐based studies at the human‐animal interface that can capture mild or asymptomatic cases. Furthermore, the burden of AI among exposed humans can be better determined by enhancing surveillance for AI at the human‐animal interface. This would be achieved by developing a region‐wide plan to enhance surveillance for AI in poultry‐exposed human populations and establishing systematic, active, and prospective AI animal surveillance programs focused on all HPAI and LPAI viruses. This should be complimented by increased event‐based surveillance in the animal health sector with robust multi‐disciplinary response to suspected AI outbreaks, including testing of exposed poultry‐workers, tracing their contacts, and assessing their seroconversion.

A region‐wide AI research agenda should be developed focused on epidemiological research and routes of transmission of AI across species. Regional training activities focused on designing and conducting AI research and surveillance should be provided. Enhancing laboratory capacity is also needed. Training on laboratory detection of AI (molecular and culture), serological detection of AI antibodies, and AI full‐genome sequencing and analysis should be conducted by both animal and public health stakeholders. Moreover, it is of utmost importance to provide training on frameworks of adapting research findings into animal and public health policies.

A tripartite WHO/OIE/FAO program is required for the region to ensure that all implemented projects are conducted with the aim of improving countries' capacities to prevent, detect, and respond to AI. One Health paradigm needs to be operationalized and institutionalized to assure cross‐sectoral communication, joint or linked surveillance systems, joint response activities, joint research activities, comprehensive national AI preparedness plans, and comprehensive national prevention, detection, and response policies.

This analysis revealed that AI research response remains weak in the EMR. To move forward, we recommend mapping and enhancing surveillance, research, and reporting capacities, improving surveillance at the human‐animal interface, and working to operationalize and institutionalize the One Health paradigm for zoonotic influenza.

## AUTHOR CONTRIBUTIONS

*Conceptualization*: Abdinasir Abubakar, Ghazi Kayali, and Wasiq Khan. *Data curation*: Rebecca Badra. *Formal analysis*: Rebecca Badra and Ghazi Kayali. Funding acquisition: Abdinasir Abubakar and Wasiq Khan. *Methodology*: Rebecca Badra and Ghazi Kayali. *Project administration*: Ghazi Kayali and Wasiq Khan. Resources: Abdinasir Abubakar and Wasiq Khan. *Supervision*: Abdinasir Abubakar, Ghazi Kayali, and Wasiq Khan. *Validation*: Stefano Tempia, Noore Alam, and Hala Abou ElNaja. *Writing – original draft preparation*: Rebecca Badra and Ghazi Kayali. Writing – review & editing: Rebecca Badra, Abdinasir Abubakar, Stefano Tempia, Noore Alam, Hala Abou ElNaja, Ghazi Kayali, and Wasiq Khan.

## CONFLICT OF INTEREST STATEMENT

The authors declare no competing interest.

## Supporting information


**Figure S1.** Search methodology flowchart.

**Figure S2.** HPAI and LPAI outbreaks in animals per year.

**Figure S3.** Records by year.

**Figure S4.** Records by country.

**Figure S5.** Records by category.

**Figure S6.** AI virus research response in Egypt, 2011–2021.

**Figure S7.** AI virus sequence submission by subtype.

**Figure S8.** AI virus sequence submission by protein.

**Figure S9.** Human influenza activity in the EMR: number of positive specimens according to virus subtype, 2011 —2021.

**Figure S10.** Human influenza activity in Egypt: number of positive specimens according to virus subtype, 2011–2021.

## Data Availability

The data that support the findings of this study are available from the corresponding author upon reasonable request.

## References

[1] Horimoto T , Kawaoka Y . Pandemic threat posed by avian influenza a viruses. Clin Microbiol Rev. 2001;14(1):129‐149. doi:10.1128/cmr.14.1.129-149.2001 published Online First: 2001/01/09 11148006 PMC88966

[2] Ducatez MF , Webster RG , Webby RJ . Animal influenza epidemiology. Vaccine. 2008;26(Suppl 4):D67‐D69. doi:10.1016/j.vaccine.2008.07.064 [published Online First: 2009/02/21] 19230163 PMC2735110

[3] FAO . Avian influenza: Telemetry studies 2021 [Available from: http://www.fao.org/avianflu/en/wildlife/sat_telemetry.htm

[4] Peiris JS , de Jong MD , Guan Y . Avian influenza virus (H5N1): a threat to human health. Clin Microbiol Rev. 2007;20(2):243‐267. doi:10.1128/cmr.00037-06 published Online First: 2007/04/13 17428885 PMC1865597

[5] CDC . Highly Pathogenic Asian Avian Influenza A(H5N1) Virus 2018 [Available from: https://www.cdc.gov/flu/avianflu/h5n1-virus.htm

[6] WHO . Avian influenza [Available from: http://www.emro.who.int/health-topics/avian-influenza/Page-1.html

[7] WHO . Cumulative number of confirmed human cases for avian influenza A(H5N1) reported to WHO, 2003‐2021 2021 [Available from: https://www.who.int/publications/m/item/cumulative-number-of-confirmed-human-cases-for-avian-influenza-a(h5n1)-reported-to-who-2003-2021-15-april-2021

[8] Hill NJ , Smith LM , Muzaffar SB , et al. Crossroads of highly pathogenic H5N1: overlap between wild and domestic birds in the Black Sea‐Mediterranean impacts global transmission. *Virus* . Evolution. 2020;7(1):veaa093. doi:10.1093/ve/veaa093 PMC7947991 34956648

[9] Hagag NM , Yehia N , El‐Husseiny MH , et al. Molecular epidemiology and evolutionary analysis of avian influenza a(H5) viruses circulating in Egypt, 2019‐2021. Viruses. 2022;14(8):1758. doi:10.3390/v14081758 [published Online First: 2022/08/27] 36016379 PMC9415572

[10] Selim AA , Erfan AM , Hagag N , et al. Highly pathogenic avian influenza virus (H5N8) clade 2.3.4.4 infection in migratory birds, Egypt. Emerging Infect Dis. 2017;23(6):1048‐1051. doi:10.3201/eid2306.162056 PMC5443452 28518040

[11] Kandeil A , Moatasim Y , El Taweel A , et al. Genetic and antigenic characteristics of highly pathogenic avian influenza a(H5N8) viruses circulating in domestic poultry in Egypt, 2017‐2021. Microorganisms. 2022;10(3):595. doi:10.3390/microorganisms10030595 [published Online First: 2022/03/27] 35336170 PMC8948635

[12] El Romeh A , Zecchin B , Fusaro A , et al. Highly pathogenic avian influenza H5N1 clade 2.3.2.1c virus in Lebanon, 2016. Avian Dis. 2017;61(2):271‐273. doi:10.1637/11544-113016-Case.1 28665732

[13] Kayali G , Webby RJ , Samhouri D , Mafi AR , Bassili A . Influenza research in the eastern Mediterranean region: the current state and the way forward. Influenza Other Respi Viruses. 2013;7(6):914‐921. doi:10.1111/irv.12136 published Online First: 2013/07/03 PMC4634261 23809648

[14] Butt KM , Smith GJ , Chen H , et al. Human infection with an avian H9N2 influenza a virus in Hong Kong in 2003. J Clin Microbiol. 2005;43(11):5760‐5767. doi:10.1128/jcm.43.11.5760-5767.2005 published Online First: 2005/11/08 16272514 PMC1287799

[15] Gomaa MR , El Rifay AS , Abu Zeid D , et al. Incidence and Seroprevalence of avian influenza in a cohort of backyard poultry growers, Egypt, august 2015‐march 2019. Emerg Infect Dis. 2020;26(9):2129‐2136. doi:10.3201/eid2609.200266 published Online First: 2020/08/21 32818403 PMC7454077

[16] Peacock THP , James J , Sealy JE , et al. A global perspective on H9N2 avian influenza virus. Viruses. 2019;11(7):620. doi:10.3390/v11070620 [published Online First: 2019/07/10] 31284485 PMC6669617

[17] Khan W , El Rifay AS , Malik M , et al. Influenza research in the eastern Mediterranean region: a review. Oman Med J. 2017;32(5):359‐364. doi:10.5001/omj.2017.70 published Online First: 2017/10/14 29026466 PMC5632697

[18] Jin H , Wang W , Yang X , et al. Evolution of H9N2 avian influenza virus in embryonated chicken eggs with or without homologous vaccine antibodies. BMC Vet Res. 2018;14(1):71. doi:10.1186/s12917-018-1391-6 29510698 PMC5840701

[19] Kandeil A , Hicks JT , Young SG , et al. Active surveillance and genetic evolution of avian influenza viruses in Egypt, 2016‐2018. Emerg Microbes Infect. 2019;8(1):1370‐1382. doi:10.1080/22221751.2019.1663712 published Online First: 2019/09/19 31526249 PMC6758608

[20] Nuñez IA , Huang Y , Ross TM . Next‐generation computationally designed influenza hemagglutinin vaccines protect against H5Nx virus infections. Pathogens. 2021;10(11):1352. doi:10.3390/pathogens10111352 34832509 PMC8625041

[21] Refaey S , Azziz‐Baumgartner E , Amin MM , et al. Increased number of human cases of influenza virus a(H5N1) infection, Egypt, 2014‐15. Emerg Infect Dis. 2015;21(12):2171‐2173. doi:10.3201/eid2112.150885 published Online First: 2015/11/20 26584397 PMC4672432

[22] Zhang AJ , To KK , Tse H , et al. High incidence of severe influenza among individuals over 50 years of age. Clin Vaccine Immunol: CVI. 2011;18(11):1918‐1924. doi:10.1128/cvi.05357-11 published Online First: 2011/09/09 21900532 PMC3209033

[23] Lohiniva AL , Dueger E , Talaat M , et al. Poultry rearing and slaughtering practices in rural Egypt: an exploration of risk factors for H5N1 virus human transmission. Influenza Other Respi Viruses. 2013;7(6):1251‐1259. doi:10.1111/irv.12023 [published Online First: 2012/11/14] PMC4634263 23145955

[24] Chinnery PF , Pearce JJ , Kinsey AM , Jenkinson JM , Wells G , Watt FM . How COVID‐19 has changed medical research funding. Interface Focus. 2021;11(6):20210025. doi:10.1098/rsfs.2021.0025 [published Online First: 2021/12/28] 34956595 PMC8504879

