# Supplementary material for: State and situation of avian influenza in the Eastern Mediterranean Region
Source: Influenza Other Respir Viruses. 2023 Apr 23;17(4):e13137. doi: 10.1111/irv.13137 (PMC10123394; doi:10.1111/irv.13137)
Supplement: Supplementary file 1 — Figure S1. Search methodology flowchart. [file IRV-17-e13137-s010.docx]

PubMed search terms “Avian Influenza” AND “country name”

561 publications retained and exported to an excel spreadsheet for abstract revision

Result: 647 publications exported to Endnote X8 library

86 duplicates were removed

21 records not related to avian influenza research in the EMR were excluded

540 relevant publications were retained

Records were collected by year, country, and category

Virology (366 papers)

Editorial (5 papers)

Epidemiology (19 papers)

Epizootiology (11 papers)

Health Care Management (23 papers)

Modeling (31 papers)

Reporting (3 papers)

Review (27 papers)

Surveillance (55 papers)
