# Supplementary figures and images for: State and situation of avian influenza in the Eastern Mediterranean Region
Source: Influenza Other Respir Viruses. 2023 Apr 23;17(4):e13137. doi: 10.1111/irv.13137 (PMC10123394; doi:10.1111/irv.13137)

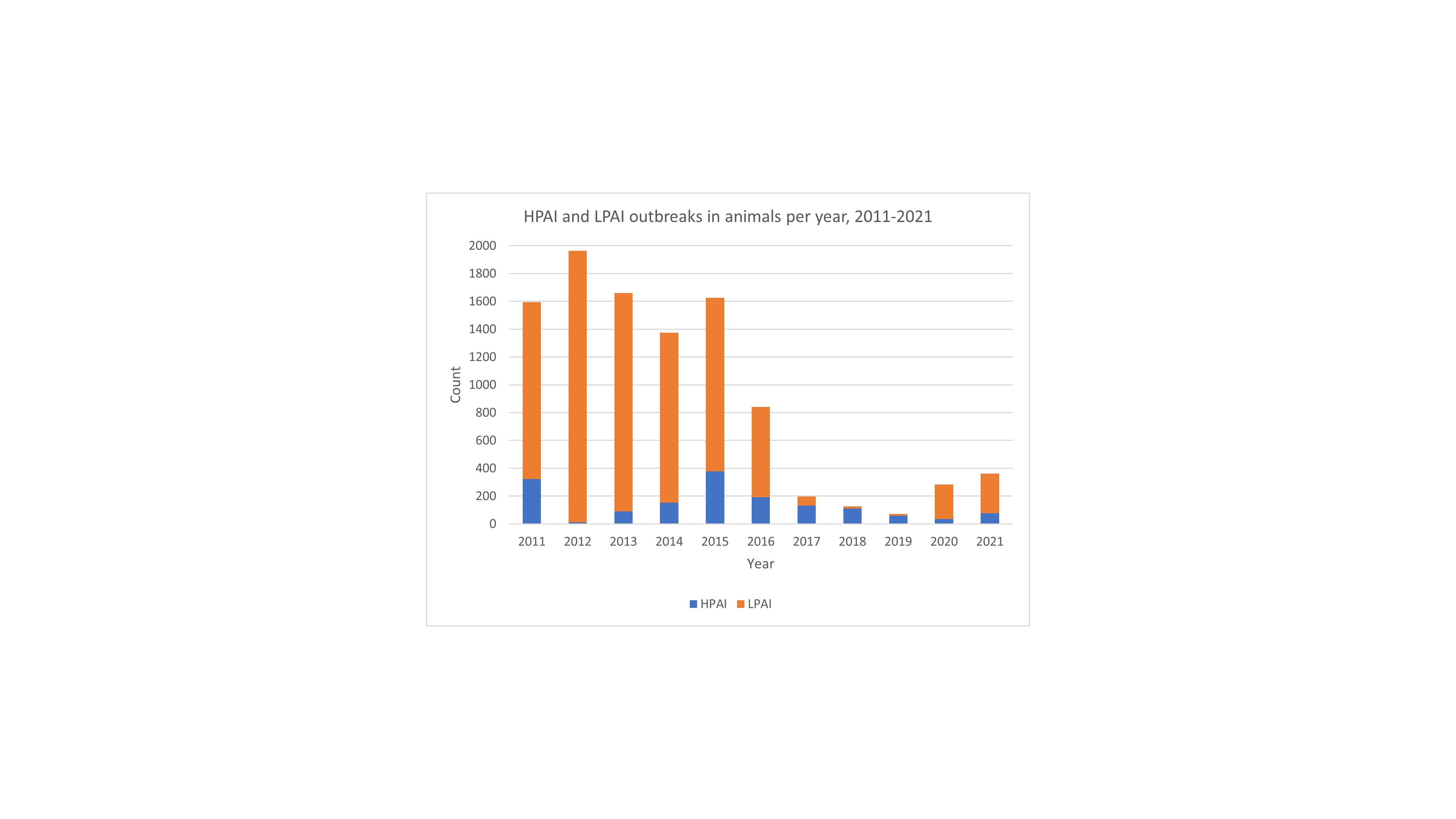

Supplement: Supplementary file 2 — Figure S2. HPAI and LPAI outbreaks in animals per year. [file IRV-17-e13137-s001.tif]

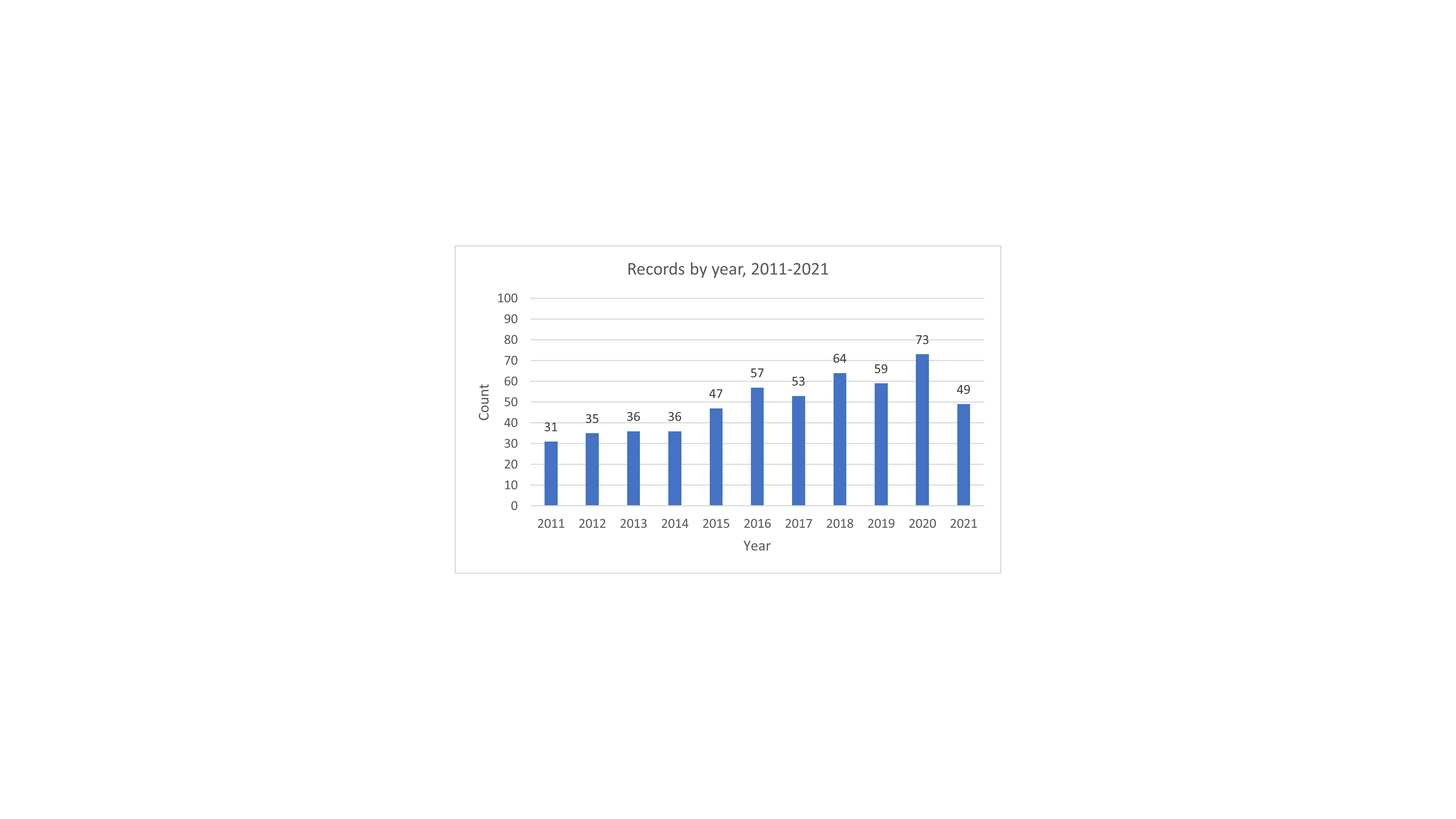

Supplement: Supplementary file 3 — Figure S3. Records by year. [file IRV-17-e13137-s006.tif]

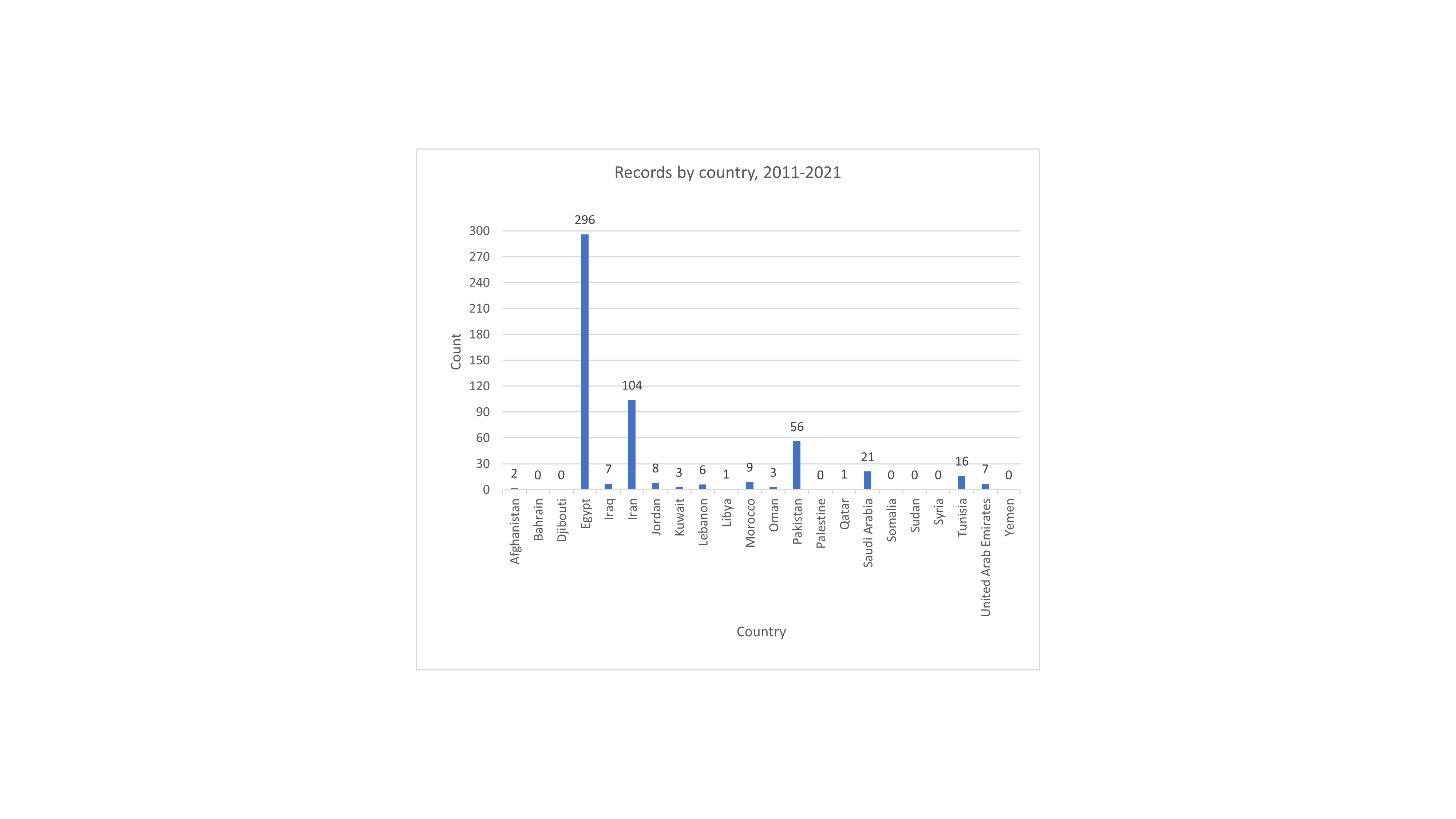

Supplement: Supplementary file 4 — Figure S4. Records by country. [file IRV-17-e13137-s004.tif]

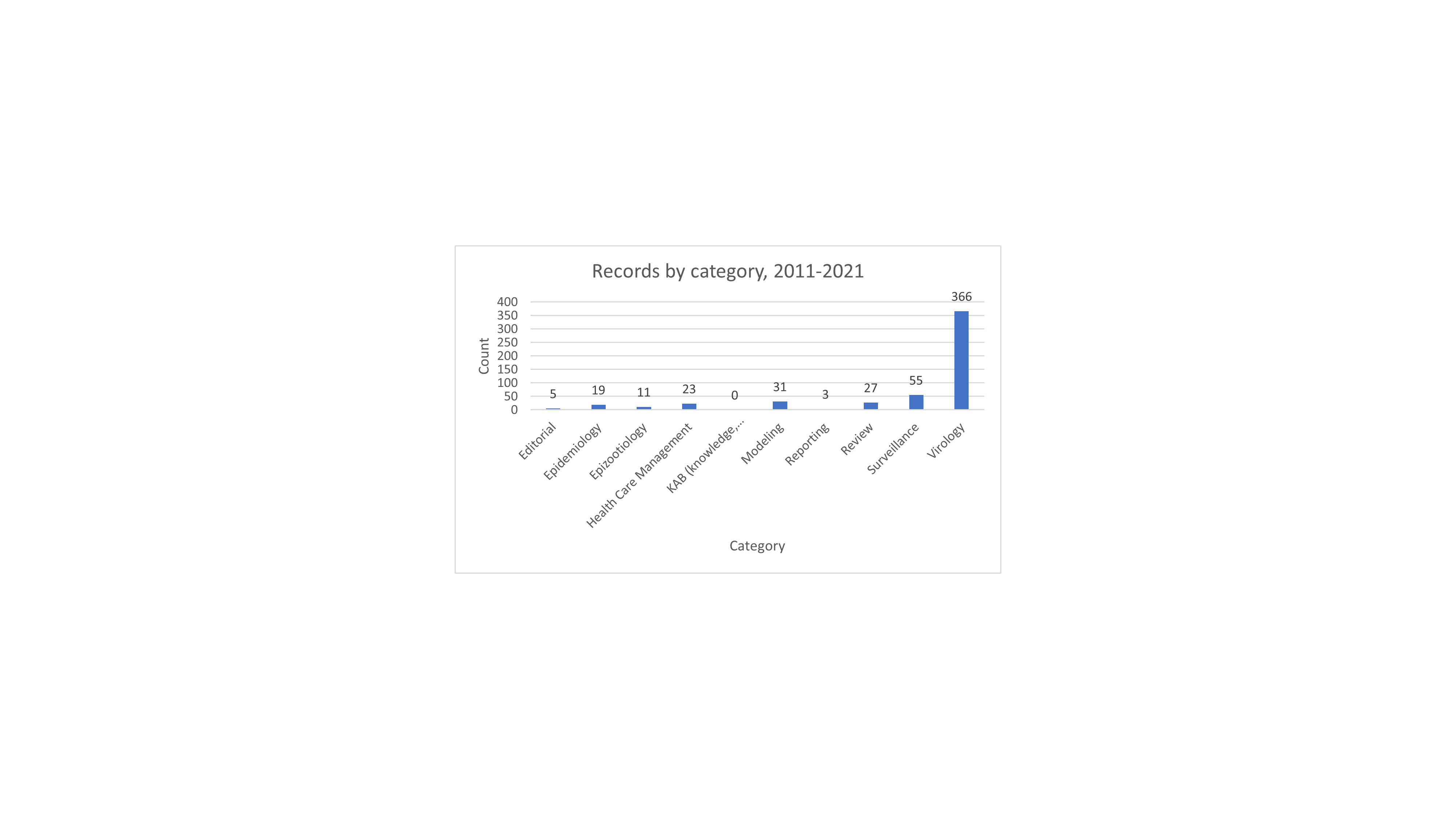

Supplement: Supplementary file 5 — Figure S5. Records by category. [file IRV-17-e13137-s009.tif]

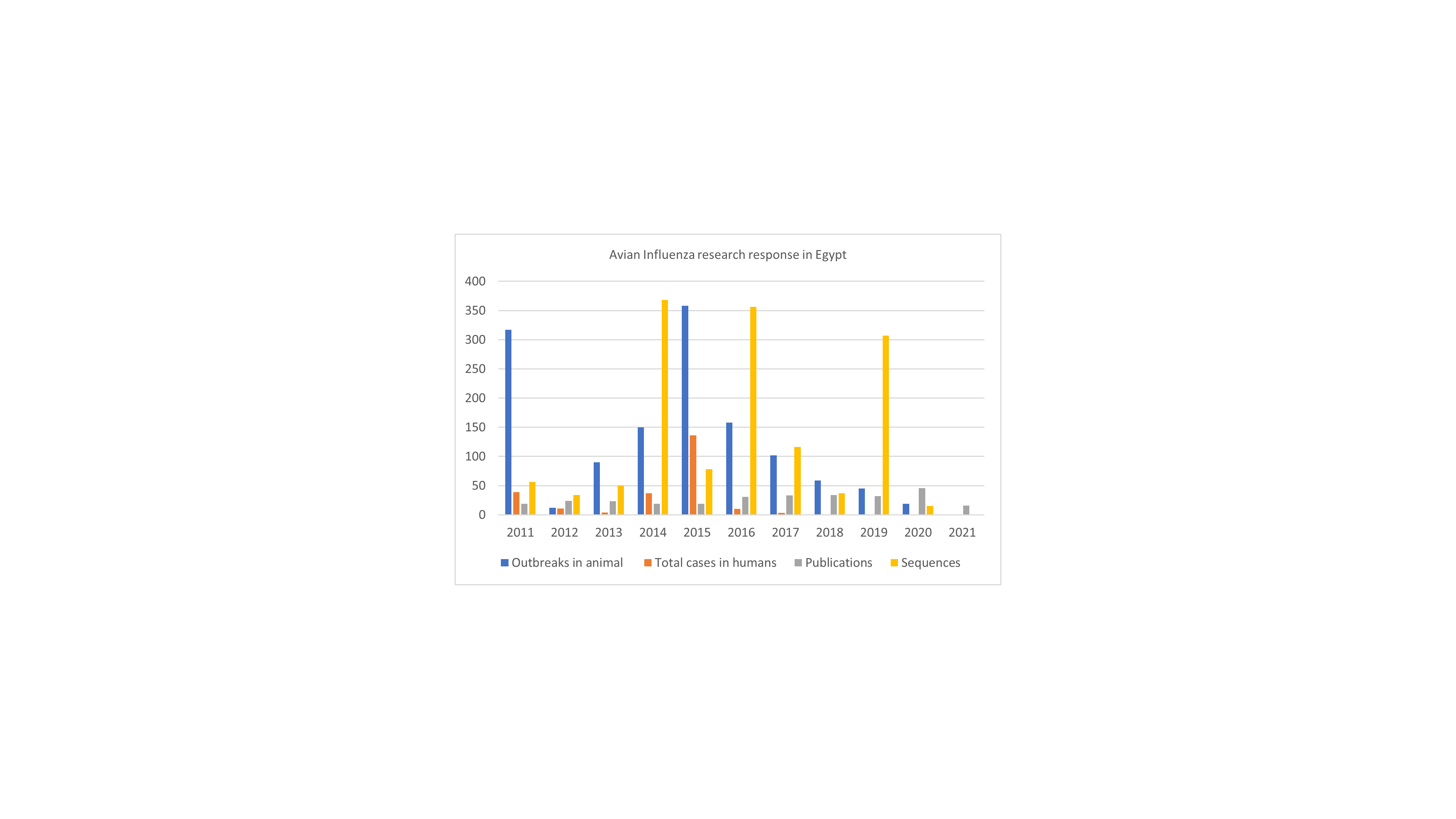

Supplement: Supplementary file 6 — Figure S6. AI virus research response in Egypt, 2011–2021. [file IRV-17-e13137-s007.tif]

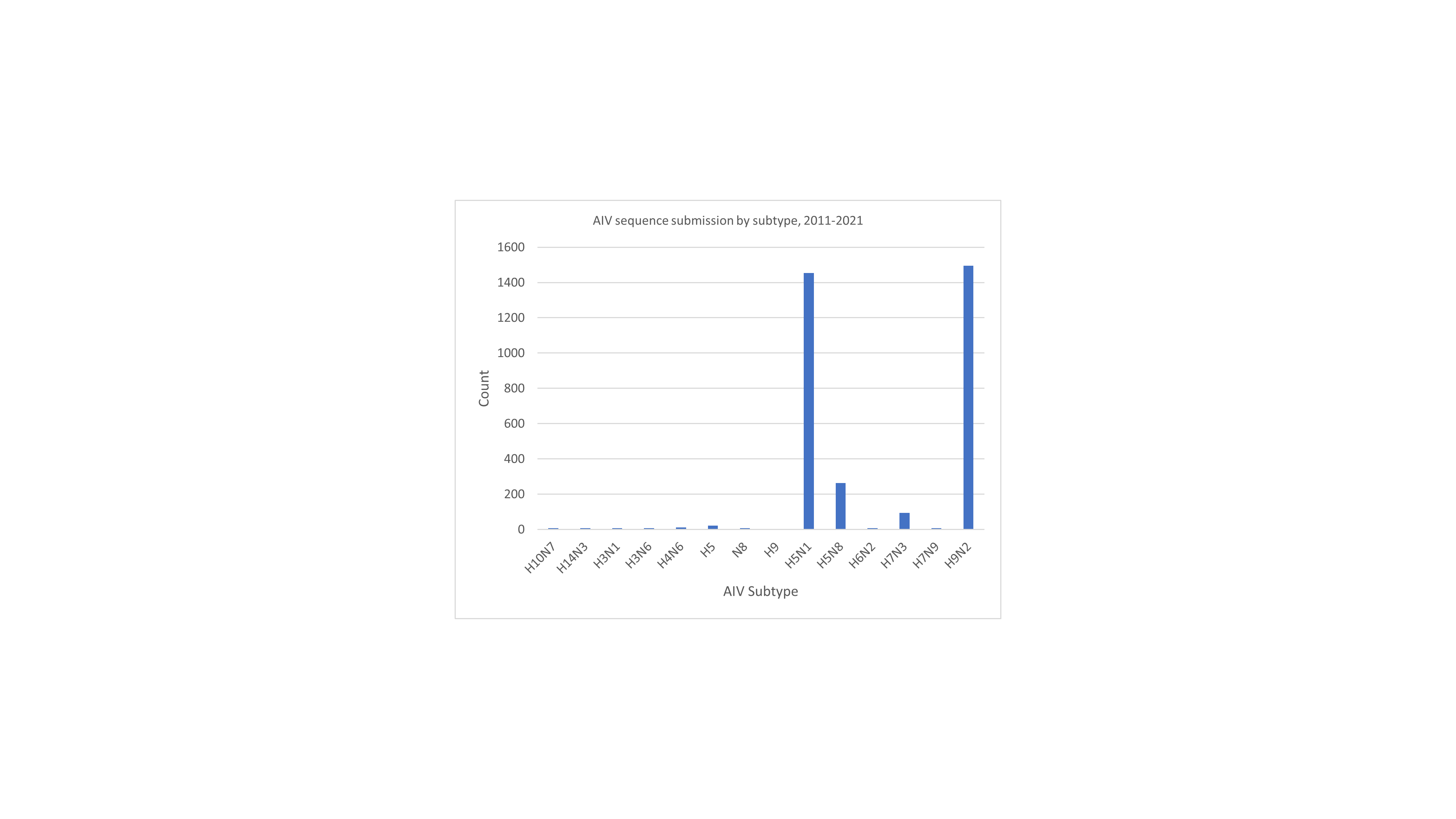

Supplement: Supplementary file 7 — Figure S7. AI virus sequence submission by subtype. [file IRV-17-e13137-s002.tif]

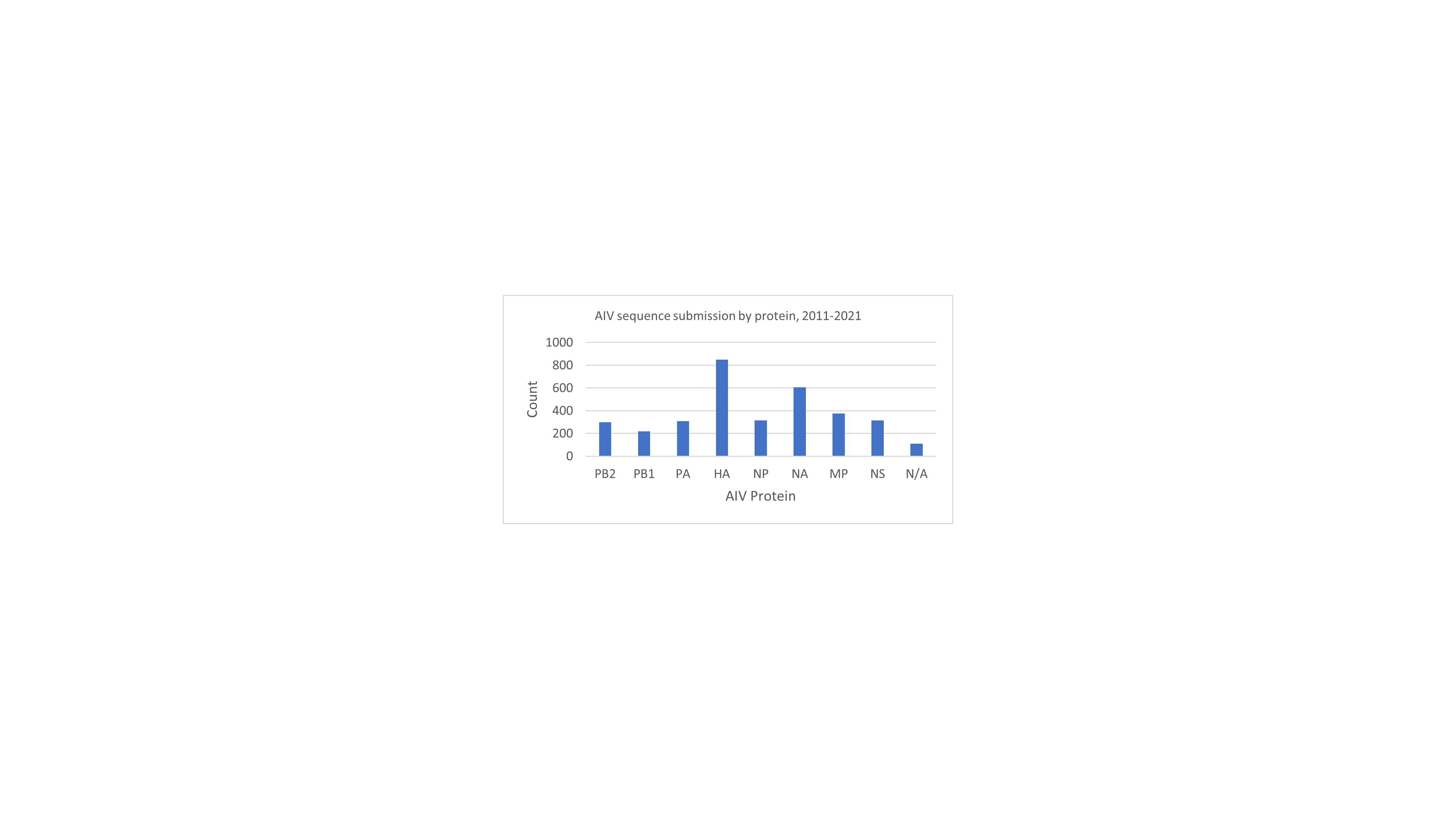

Supplement: Supplementary file 8 — Figure S8. AI virus sequence submission by protein. [file IRV-17-e13137-s008.tif]

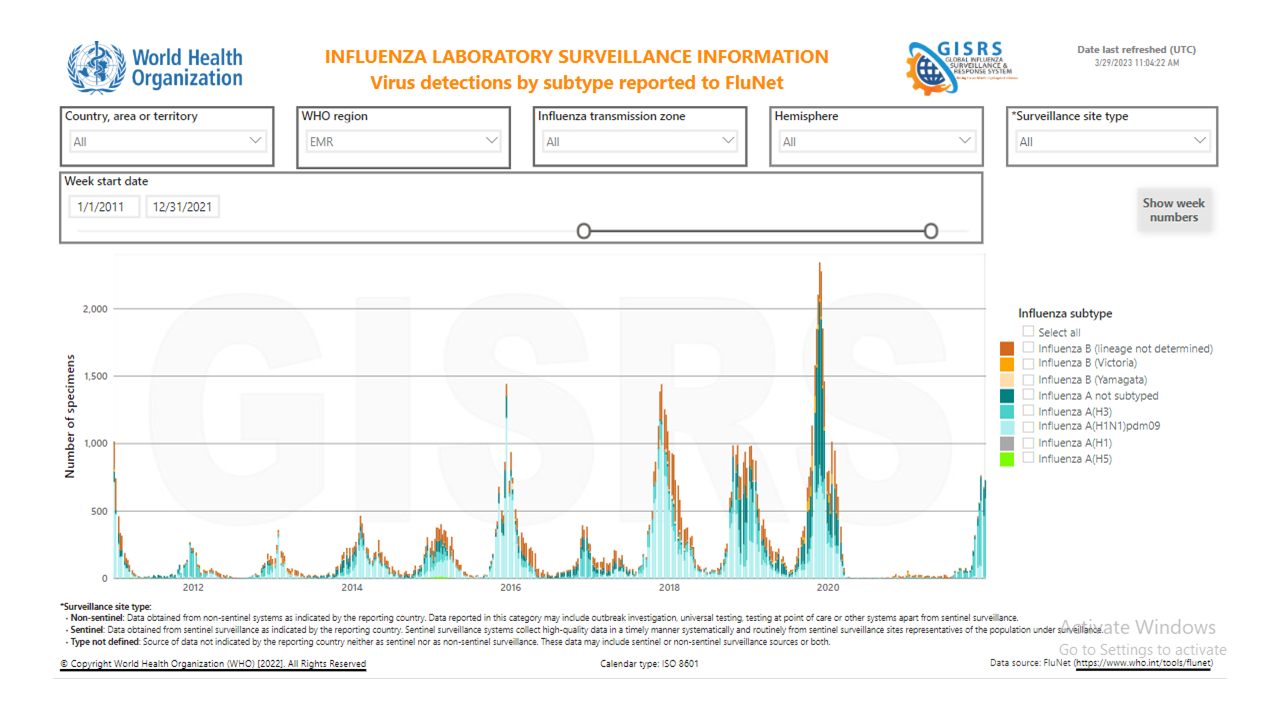

Supplement: Supplementary file 9 — Figure S9. Human influenza activity in the EMR: number of positive specimens according to virus subtype, 2011 —2021. [file IRV-17-e13137-s005.tif]

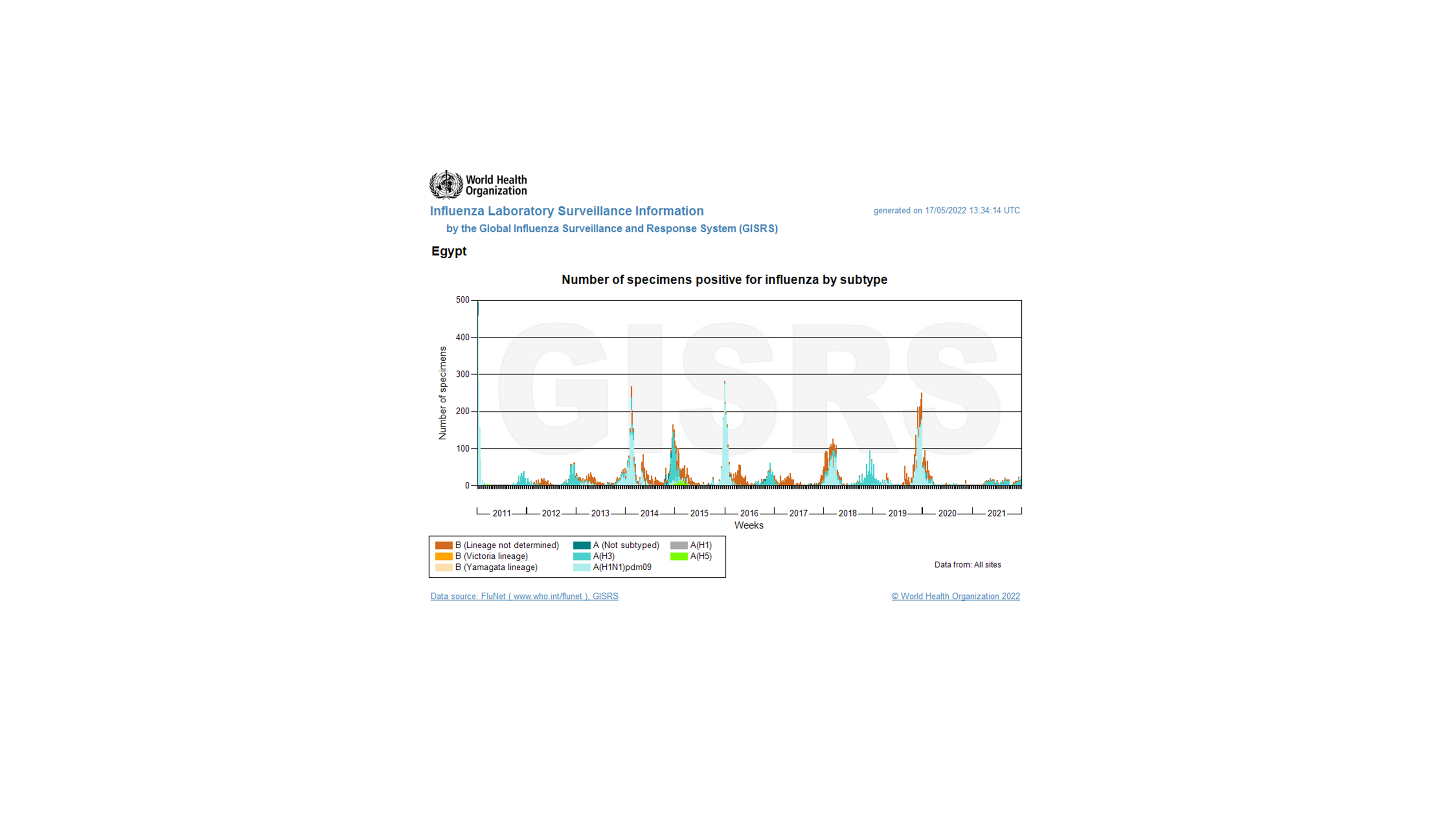

Supplement: Supplementary file 10 — Figure S10. Human influenza activity in Egypt: number of positive specimens according to virus subtype, 2011–2021. [file IRV-17-e13137-s003.tif]
